# Successful Ablation of Antero-septal Accessory Pathway in the Non-Coronary Cusp in a Child

**DOI:** 10.1016/s0972-6292(16)30506-x

**Published:** 2012-05-20

**Authors:** Daisuke Kobayashi, Swati O Arya, Harinder R Singh

**Affiliations:** Division of Pediatric Cardiology, Children's Hospital of Michigan, The Carman and Ann Adams Department of Pediatrics, Wayne State University School of Medicine, Detroit, Michigan

**Keywords:** Non-coronary cusp, child, antero-septal accessory pathway, Wolff-Parkinson-White syndrome, ablation

## Abstract

A 15-year-old boy with Wolff-Parkinson-White syndrome underwent an electrophysiology study for symptoms of palpitations and persistence of pre-excitation during peak exercise. He was detected to have right antero-septal accessory pathway with relatively long effective refractory period and no inducible tachycardia. He had only transient normalization with cryoablation. Eight months later, he presented again with two episodes of seizures with preceding palpitations. Neurology evaluation was unremarkable with a normal electroencephalogram. In view of his symptoms in association with evidence of pre-excitation, he underwent a second electrophysiology study with ablation. Cryoablation in the anterior septum again achieved only transient normalization. Mapping in the non-coronary cusp identified an earliest accessory pathway potential. RF ablation was performed in the non-coronary cusp with immediate normalization of his electrocardiogram. At 6 month follow-up, he continues to have no pre-excitation on his EKG. Ablation of the anteroseptal accessory pathway in the non-coronary cusp can be safely performed in patients' refractory to conventional ablation sites and techniques.

## Introduction

Catheter ablation is well established as a definitive therapy of accessory pathways (AP) in children. However, antero-septal and mid-septal AP remains a challenging target due to the anatomic proximity to the normal conduction system. Recurrence rate and risk of atrioventricular (AV) block is higher in patients with septal fibers. Successful ablation of anteroseptal AP in the non-coronary cusp (NCC) has been reported [1,2]. The experience in this novel ablation technique is still evolving. We report a child in which the anteroseptal AP was successfully treated with catheter ablation in the NCC.

## Case Report

A previously healthy 15-year-old boy was referred because of palpitations and pre-excitation on electrocardiogram (ECG). He had a structurally normal heart. The ECG showed evidence of Wolff-Parkinson-White syndrome. Treadmill exercise stress test revealed persistent pre-excitation at peak exercise (maximal heart rate of 190 beats per minute). Initial electrophysiology (EP) study revealed normal sinus and AV node function. AP effective refractory period was detected to be 600/380 msec. Atrial pacing protocol did not induce any tachyarrhythmia with and without isoprenaline. Mapping in sinus rhythm revealed antero-septal location of the accessory pathway. Cryoablations performed in the right anterior septum resulted in transient near normalization of ECG but failed to eliminate the pre-excitation. Further ablations were aborted after discussions with the family about the relatively long AP effective refractory period with no inducible tachyarrhythmia and higher potential risk of damage to the AV node.

**Figure 1:**
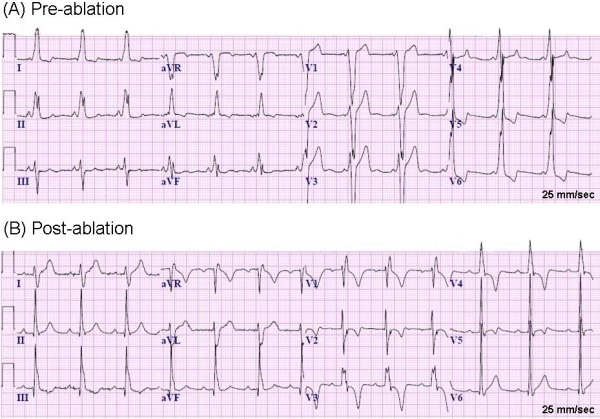
Twelve lead Electrocardiogram. (A) Pre-ablation: There is an evidence of pre-excitation characterized by a delta wave and short P-R interval of 95 msec, wide QRS. The overall delta wave axis is consistent with a right antero-septal accessory pathway. However, a small positive delta wave is seen in lead V1 and the delta wave in lead III is less positive than the delta wave in lead II. (B) Post-ablation. Note the normal PR interval of 195 msec and disappearance of delta wave. There is an evidence of right bundle branch block with QRS duration of 117 msec and QRS frontal plane axis of 80º.

Eight months later, he presented with two episodes of seizures with preceding sensed tachycardia. His electroencephalogram was normal and the events were thought to be non-epileptic per neurology. His ECG showed persistent preexcitation (Figure 1A). He underwent a repeat EP study to eliminate pre-excitation from the potential etiologies of seizures.

**Figure 2:**
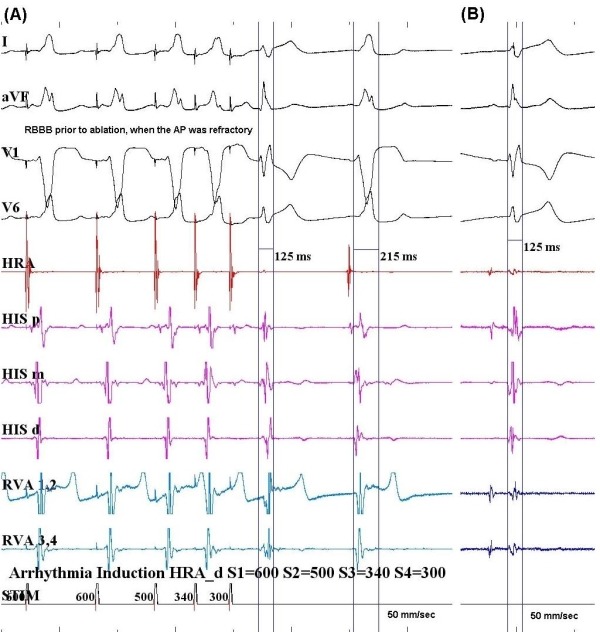
(A) Surface and intracardiac electrocardiograms with arrhythmia induction with 600/500/340/300 msec. The right bundle branch block (QRS duration 125 ms) was noted prior to the ablation, with the same axis and configuration as seen following ablation. (B) Surface and intracardiac electrocardiograms following ablation. There is no change in QRS axis, morphology and duration as compared to pre-ablation QRS with accessory pathway being refractory.

**Figure 3:**
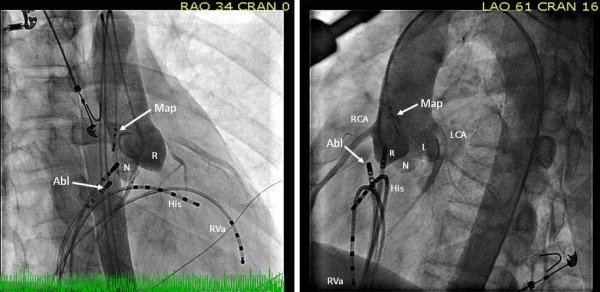
Aortogram obtained in the right anterior oblique and left anterior oblique projections shows the target ablation sites. (Abl, cryoablation catheter (right atrial approach); Map, mapping catheter (non-coronary cusp approach); His, His-bundle electrogram; RVa, right ventricular apex; RCA, right coronary artery; LCA, left coronary artery; R, right coronary cusp; L, left coronary cusp; N, noncoronary cusp.)

**Figure 4:**
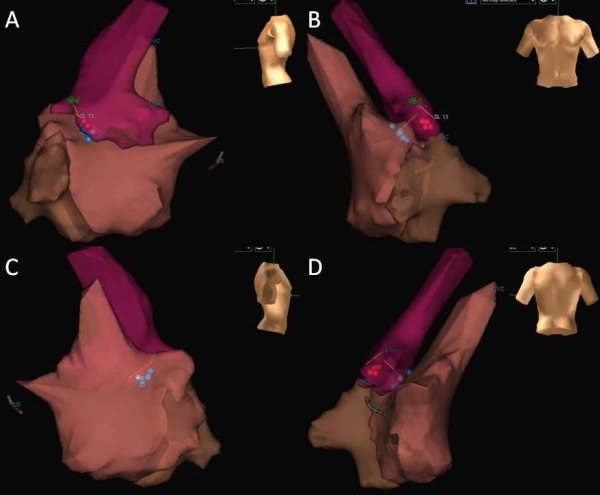
Three-dimensional mapping of right atrium (orange), right ventricle (brown) and aortic root (purple) from different projection: (A) left lateral, (B) front, (C) right lateral, and (D) back. Blue dots represent sites of cryoablation from the right atrium and right ventricle with transient normalization and red dots represent sites of radiofrequency ablation in the non-coronary cusp.

**Figure 5:**
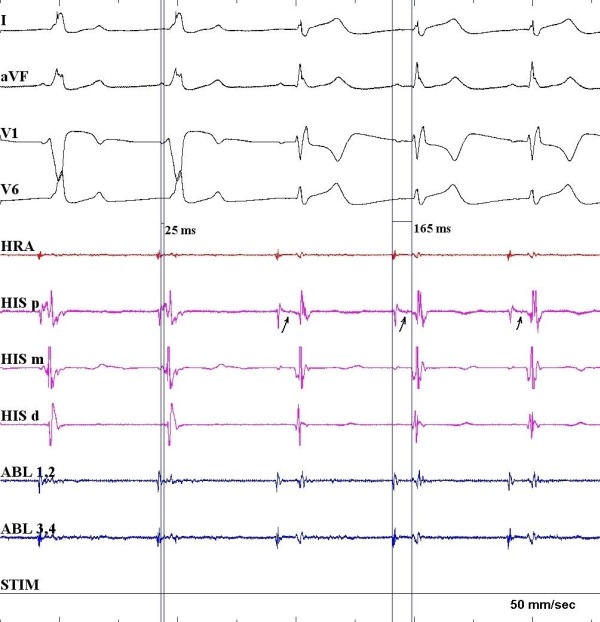
Ablation of a right anteroseptal accessory pathway in a non-coronary cusp. Application of radiofrequency ablation results in loss of pre-excitation. The earliest ventricular signal on ablation catheter preceded the delta wave by 25 ms. His signal (arrow) is noted on the proximal His tracing after the ablation.

Repeat EP study revealed normal sinus and AV node function. Atrial pacing protocol did not induce any tachyarrhythmia with and without isoprenaline. Mapping in sinus rhythm showed evidence of antero-septal AP as discovered in the previous EP study. Right bundle branch block (RBBB) pattern was noted pre-ablation when the AP was refractory (Figure 2A). Cryoablations were performed in the right anterior septum with transient near normalization of ECG as was seen in the previous attempt. Mapping was then performed in the aortic root using a St. Jude's 4-Fr deflectable Inquiry catheter after administering heparin to maintain activated clotting time above 250 sec and obtaining an aortic root angiogram to delineate the aortic root anatomy and origin of the coronary arteries (Figure 3). Aortic root anatomy was created on the 3D mapping system (Figure 4). Mapping in the non-coronary cusp identified an earliest ventricular signal on ablation catheter preceding the delta wave by 25 ms (Figure 5). A 7-Fr Blazer Radiofrequency ablating catheter was advanced retrogradely to the aortic root. The distance of the coronary orifice to the site of earliest ventricular activity was measured to be 1.4 cm. Radiofrequency ablation was performed using 20 Watts energy with target tissue temperature of 50º C in the non-coronary cusp with immediate normalization of ECG within 4 secs (Figure 5) with no pre-excitation, normal PR interval (165 ms) and RBBB pattern (Figure 2B). Intravenous adenosine was administered to obtain transient AV block and document no residual accessory conduction. No pre-excitation was seen after waiting for one hour. No tachyarrhythmia could be induced with an aggressive atrial pacing protocol without and with isoprenaline. At 6 month follow-up, his EKG continues to show no pre-excitation with RBBB pattern, QRS frontal plane axis 80º with first degree AV block. Exercise stress test showed that PR interval shortened to 120 ms with heart rate above 150 beats per minute, as compared to PR interval of 150 ms with heart rate of 100 beats per minute with no change in QRS duration, axis and morphology with no inducible tachyarrhythmia.

## Discussion

Ablation of anteroseptal AP remains challenging because of its close proximity to normal conduction system and the potential risk of heart block. Acute success rate for RF ablation of anteroseptal AP is 96% in the current era. However the recurrence rates and risks of AV block are higher in the antero-septal AP. Cryoablation can be applied to septal AP to avoid injury to the normal conduction system but its success rate is suboptimal [3].

Antero-septal AP has been successfully eliminated by RF and cryo-ablation in the NCC on rare occasions [1,2]. In patients with previously failed ablation, careful mapping of an antero-septal AP in the NCC has been suggested as an alternative approach [1]. The NCC has immediate anatomic relationship with the interatrial septum and the septal portions of the right and left atrium. Possible electrically active myocardial connections, constituting an AP, can occur in the region of NCC between ventricular myocardium (just below and extending above the NCC) and atrial myocardium (adjacent interatrial septum). Non-coronary cusp ablation has been reported in young adolescents and adults as well as a 4-month-old infant with incessant orthodromic atrioventricular reentrant tachycardia [2]. Huang et al reported the use of irrigated RF ablation in the NCC for ablating antero-septal AP, which could not be ablated despite 3 ablation procedures in the right and left atria, and that recurred after successful ablation in the NCC with conventional RF energy [1]. Irrigated energy in the NCC may be an alternative approach in patients with failed conventional energy.

A small positive delta wave occurring in lead V1 and a delta wave in lead III that is relatively isoelectric or much less positive than the delta wave in lead II has been reported to distinguish with the typical antero-septal AP. Interestingly, these ECG features can be seen in our case.

Complications such as coronary artery occlusion have been reported in cases of RF energy delivery through the aortic sinus of Valsalva [4]. To avoid complications associated with RF ablation in coronary cusps, it is advocated that the distance between the ablation catheter tip and the ostium of the left and right coronary artery should be more than 1.0 cm before RF energy is applied, and the tip temperature should be maintained below 55 Cº to avoid perforating the sinus of Valsalva, which would damage the aortic valve and cause coronary occlusion [4]. We noticed RBBB following ablation of the AP. However on reviewing the recordings it was noted that he had similar morphology and QRS axis in the beats where the AP was refractory prior to ablation. Therefore it could be concluded that it was not a complication related to the ablation in the NCC. The first degree AV block seen on follow up could be related to multiple causes like multiple ablations in the antero-septum in the first and second EP study, ablation in the NCC or hypervagotonia. His PR interval after ablation was noted to be 165 ms however on follow up was detected to have first degree AV block possibly implying effect of vagal tone or delayed effect of ablation. His PR interval shortened to 120 ms with a heart rate >150 beats per minute during the exercise stress test. This favors effect of vagal tone as the etiology of prolonged PR interval.

There is growing evidence showing a possible association between ventricular pre-excitation and ventricular dysfunction in the absence of sustained tachyarrhythmia [6]. The proposed mechanism of this association is that ventricular pre-excitation mediated left ventricular dyssynchrony may induce cardiac remodeling and dysfunction. The right-sided septal and paraseptal AP are commonly involved in patients with left ventricular dysfunction. Kwon et al reported that systolic function was reduced in patients with septal AP, compared to patients with left or right-sided free wall AP. Udink ten Cate et al reported 10 children (mean age: 8 years) presenting with dilated cardiomyopathy and overt ventricular pre-excitation in the absence of incessant tachycardia [6]. In all patients, right sided AP was confirmed and left ventricular function completely recovered after a loss of pre-excitation. Currently, EP study and ablation may be considered in patients with asymptomatic ventricular pre-excitation with strong predictors for potentially life-threatening arrhythmic events such as inducibility, anterograde refractory period of AP ≤250 msec, and multiple APs. The right sided AP may be considered as the potential indication for ablation in asymptomatic ventricular pre-excitation to prevent ventricular dysfunction. Ablation in the NCC may need to be performed for these fibers in patients that are refractory to ablation in the conventional locations.

## Conclusion

We report a successful ablation of antero-septal AP in non-coronary cusp without complications. This approach can be safely performed with adequate precautions and may be considered as an alternative in previously failed ablation for antero-septal AP.
